# Public health nurses’ experiences following up children with overweight and obesity according to national guidelines. A qualitative study

**DOI:** 10.1080/17482631.2024.2306658

**Published:** 2024-01-23

**Authors:** Hanna Skjelbred Nygaard, Kirsten Gudbjørg Øen

**Affiliations:** Department of Public Health, Faculty of Health Sciences, University of Stavanger, Stavanger, Norway

**Keywords:** Children, overweight, obesity, guidelines, public health nurse, school nurse, communication, qualitative, Hildegard Peplau

## Abstract

**Purpose:**

This study aimed to develop knowledge of how the follow-up regarding overweight and obesity among children in primary school is experienced by the PHN and how the guidelines may be used to improve health services in this follow-up.

**Methods:**

We analysed semi-structured interviews of 9 PHNs using qualitative content analysis.

**Results:**

Two themes emerged: Following up with children with overweight and obesity is an important but challenging duty; The PHNs call for clearer guidelines. Following five sub-themes: PHNs strive to adhere to the guidelines, show compassion in the follow-up, have difficulty handling parents’ feelings and reactions, feel alone with the responsibility, and have suggestions for clearer guidelines.

**Conclusions:**

PHNs call for enough resources to communicate the results of the child’s weight in a sufficient form. PHNs and families should establish common goals. The PHN should avoid one-way communication but meet the parents’ concerns and needs. This requires the PHN to focus on building a secure relation to the child and the families, as described by Peplau. Guidelines must include instructions and tools on how to communicate and meet the family’s concerns. Political action and increased funding could strengthen the follow-up and thereby prevent more obesity among children, which can be a predictor of poorer health outcomes later in life.

## Introduction

Worldwide, the number of people with obesity has nearly tripled since 1975. In 2016, 39% of adults 18 and older were overweight, and 13% were obese (World Health Organization, 2021). Among Norwegian children, 17% of those aged 6–11 are overweight or obese (Júlíusson et al., 2010). Overweight and obesity are defined based on the child’s body mass index (BMI). Children have reduced skeletal- and muscle mass before puberty, and girls are developed earlier than boys. Therefore, it is recommended to use ISO-BMI when defining a child’s weight, which is their weight adjusted for gender and age. Overweight is defined as ISO-BMI ≥25 and obesity as ISO-BMI ≥30 (Cole et al., 2000). Obesity in childhood and adolescence increases the risk of becoming overweight or obese in adulthood (Singh et al., 2008). Childhood body mass index (BMI) predicts the risk of prediabetes and cardiovascular disease in adulthood (Ajala et al., 2017). Severe obesity in childhood is even associated with liver complications (Seth et al., 2020). Furthermore, childhood obesity is associated with psychological comorbidities, such as depression, lower quality of life, emotional and behavioural disorders, and lower self-esteem (Rankin et al., 2016). Therefore, it is imperative to prevent children from becoming obese and to follow up with those children identified as overweight and obese to normalize weight development and promote a higher quality of life.

In Norway, the public health nurse (PHN) is a registered nurse with further education or a master’s degree in public health nursing. The focus area for the PHN is health promotion and prevention of disease among children, adolescents, and their families. PHNs works at health centres for children between 0–5 years and/or in the school health service for children and adolescents between 6–20 years and/or at health centres for young adults between 16–25 years. The PHNs work tasks are varied, and involves health examination of children/adolescents, guidance regarding breastfeeding and dietary, infection prevention, vaccination, sexual education, guidance regarding contraception, health talks etc. (Andersen et al., 2022).

Children with overweight and obesity are a focus area for the school health service in Norway. In 2010, the national guidelines for the prevention, assessment, and treatment of overweight and obesity among children and adolescents were published. The guidelines state that the PHN is responsible for detecting and following up on overweight and obesity among children and adolescents. In the school health service, this means measurements of height and weight in first, third, and eighth grade and otherwise when indicated. According to the national guidelines, the PHN is responsible for offering the parents and even the child a conversation about the child’s weight development. The PHN and the parents may decide whether they want the child to be present. Furthermore, if the parents want to receive follow-up from the school health service, the PHN should offer follow-up focusing on motivation to make lifestyle changes involving the whole family (Helsedirektoratet, 2010). For the implementation of guidelines to be effective and sustainable, it is essential to explore the experience from the perspective of the professionals working with the family. The health care professionals using the guidelines should therefore be an integral part of evaluating and monitoring the impact the guidelines have over time (Beauchemin et al., 2019). It is important to study the PHNs experience to further improve the guidelines and thus improve the treatment of overweight and obesity among children and adolescents.

Previous Norwegian research on the implementation of the national guidelines showed that PHNs believed that the guidelines led to better practice and improved quality and were a useful tool in the follow-up of overweight and obesity (Nordstrand et al., 2016). However, the PHNs did not feel included or involved in planning the content of the guidelines despite being responsible for the follow-up (Helseth et al., 2017). When using the guidelines, the PHNs felt there was poor interdisciplinary collaboration with other professionals, such as general practitioners (GPs), nutritionists, and physiotherapists, which made the PHNs feel alone in the follow-up (Helseth et al., 2017; Nordstrand et al., 2016). Some PHNs also felt they lacked competence about overweight and obesity. In addition, the PHNs experienced a lack of time and resources and believed that the work challenged their own attitudes and feelings (Helseth et al., 2017). According to Nordstrand et al. (2016), many PHNs found the guidelines useful but did not have time to implement them. How PHNs implemented the guidelines for obesity was affected by determinants at different organizational levels. Support from the organization and its leaders were mentioned as important to implement the guidelines (Nordstrand et al., 2016).

International studies have similar findings with Norwegian studies on the implementation of guidelines in the management of childhood overweight and obesity. Many countries recommend using guidelines on the prevention and management of childhood overweight and obesity to achieve a mutual structure, and to put into system an interdisciplinary collaboration between different health professionals. However, the barriers on implementing the guidelines are also similar in international studies such as lack of knowledge on childhood obesity, lack of dialogue between different professions, lack of time, resources and governmental support (Gooey et al., 2022; Nittari et al., 2019; Oliveira et al., 2022; Panagiotopoulos et al., 2011; Seidell & Halberstadt, 2020; Weihrauch-Blüher et al., 2018).

Obesity is a sensitive topic. Previous research has shown that parents experience conversations about overweight and obesity as stigmatizing and have not noticed that their child is overweight before the PHN addresses it (Støles et al., 2019; Thorstensson et al., 2018; Toftemo et al., 2013). The PHNs role in the follow-up of overweight and obesity may be challenging due to the responsibility to clearly communicate the results to the parents while simultaneously avoiding stigmatizing the child or the parents. According to Ames et al. (2020), the mode of communication between the PHN and the parents may affect the parents’ reaction to the results of weight screening in their child. Both the timing and content of the letter given to the parents are highlighted as important for how the parents will react. Furthermore, parents who do not believe that their child is overweight or obese despite the result are less likely to change their behaviour regarding the child’s weight (Ames et al., 2020).

When addressing a sensitive topic, like a child’s weight development, the establishment of a relation to the child and the family is important to avoid stigmatization. In addition to this, the national guidelines require the PHN to take on several roles, as giving guidance, support, listening to the patient, supply knowledge etc. These are also main elements in Hildegard Peplau’s interpersonal relation theory (Peplau, 1991) which is originally taken from the field of psychiatric nursing but is equally relevant to understanding the nurse-client relationship when it comes to the follow-up of overweight and obesity. The word “therapeutic” will be used, not because the PHN is to be considered a therapist but because the therapeutic alliance described by Peplau is important in understanding what is required to establish a good nurse-client relationship. Peplau emphasized the nurse-client relationship as the basis of nursing. To establish a therapeutic alliance between the nurse and the patient, Peplau believes two factors must be present: The patient must have a “felt need” (i.e., a health problem), and it is crucial that the patient considers the professional help available as useful (Peplau, 1991).

Peplau describes multiple roles developing through the nurse-client relationship: stranger, resource, teacher, leader, surrogate, and counsellor. Through these roles, the goal is to establish an interpersonal relationship. In the role of stranger, the nurse and patient start to build a relationship. Both parties must show mutual respect and acceptance towards each other. The nurse must listen to the patient. The resource role involves supplying knowledge and providing answers to problems. The teacher role concerns learning through experience with a focus on problem-solving, which may develop into habits for the patient. In the leadership role, the nurse should offer the patient direction. In the surrogate role, the patient may cast the nurse into other roles, such as a surrogate for mother, father, sibling, etc., based on earlier experiences and feelings. The last role is the role of counsellor, in which the nurse helps the patient to become aware of and strengthen the patient’s resources so that the patient can see what is required for health or what threatens health. The patient eventually becomes independent (Peplau, 1991).

Since the guidelines were published in 2010, increasing media reports have claimed that weighing and measuring children have caused eating disorders (Dyregrov, 2021; Vogt, 2021). Also, some health care professionals have stated in the media that weighing and measuring children are doing more harm than good. Researchers in the field have also admitted in the media that the treatment for children with obesity gives poor results and that children with obesity have too few options for follow-up (Meland et al., 2021). According to Shaharabany et al. (2020) there is a research gap on this area, especially regarding the long-term effects. However, this study did not find an increase in eating disorders after participating in a weight management programme for children and their families (Shaharabany et al., 2020). Other studies also show that there is no increase in developing eating disorder when following a weight management programme supervised by health professionals (De Giuseppe et al., 2019; House et al., 2021; Jebeile et al., 2019). According to House et al. (2021), the risk of an eating disorder should not prevent access to care for children with obesity which may lead to severe health risks for the child (House et al., 2021).

Media reports influence the attitude of everyone involved, including parents, children, and health care personnel. Many years have passed since previous studies were carried out on implementing the national guidelines in Norway. The health care service will be affected by societal developments, and the media will be an important factor influencing attitudes and behaviour. Several municipalities in Western Norway have requested more knowledge on the follow-up of overweight and obesity in children to improve the quality of the service offered. There is a gap in knowledge if the health care professionals are affected by the public published criticism of the work they carry out, and whether the criticism leads to changes in the practice of following the guidelines for follow-up of children with overweight and obesity.

The purpose of this study was to provide new knowledge from the view of health professionals working with this group of children, that may contribute as a basis for an improved health service regarding the follow-up of overweight and obesity in children and adolescents.

## Aims

This study aimed to develop knowledge of how the follow-up regarding overweight and obesity among children in primary school is experienced by the PHN and how the guidelines may be used to improve health services in this follow-up.

### Research questions

- What are the public health nurse’s experiences working with the national guidelines for the prevention, assessment, and treatment of overweight and obesity for primary school children?
- What proposals does the public health nurse have to further improve the follow-up for children with overweight and obesity based on the national guidelines?

## Materials and methods

Following Graneheim and Lundman (2004), individual qualitative interviews were chosen with qualitative content analysis to explore the meaning behind the descriptions given by the participants. According to Graneheim and Lundman, a text always implies several meanings, and there is always a certain degree of interpretation when reading a text. This method analyses both manifest and latent content (Graneheim & Lundman, 2004).

### Recruitment and sample

The study included PHNs with work experience of at least one year working in primary school. Several municipalities in Western Norway were contacted, and participants from five municipalities took part in the study. These municipalities were of different sizes. When used later in the text, the phrase “smaller municipalities” refers to a population between 1000 and 3000 inhabitants. The recruitment was initiated by contacting the leader of the school health service, who passed an email on to the PHNs working in primary schools in their area. Some PHNs made contact after receiving the email, but due to a difficult recruitment process, snowball sampling was used to recruit participants. Snowball sampling finds individuals with the desired characteristics for a study and uses their social network to recruit other participants (Sadler et al., 2010).

A sample of 9 PHNs were interviewed. According to Malterud et al. (2016), there are five items in qualitative studies that affects the studies richness and information power: The studies aim, sample specificity, use of established theory, quality of dialogue and analysis strategy. How broad the aim of the study is determining how many participants the study needs. In this study we focused on the follow-up on overweight and obesity to children in primary school, as well as narrowing it down to focus on national guidelines. To offer sufficient information power it will also depend on whether the participants have characteristics that are specific for the study’s aim. We recruited PHNs currently working with the follow-up of overweight and obesity among children in primary school. They had both the education and experience specific to answer the study’s aim. At the same time, we focused on interviewing PHNs from different municipalities of different size to provide richness in the data. Well-developed theories and analysis methods were also used throughout the study. The dialogue in the interviews is important to get sufficient information from the participants. The participants in this study provided valuable and sufficient information and answered the aim of the study. We also chose to increase with some more participants than originally planned due to new information in some interviews. We ended the interviews when the same information was repeated by the participants (Malterud et al., 2016).

### Data collection

The interviews took place from November 2021 to January 2022. Individual interviews were conducted with a semi-structured interview guide with the following questions: How do the public health nurses experience working with the national guidelines for the prevention, assessment, and treatment of overweight and obesity in children and adolescents, and which suggestions does the public health nurse have to further improve the follow-up of children with overweight and obesity based on the national guidelines? The interviewer listened carefully to the PHN’s story and used follow-up questions when relevant to get more information to answer the research questions. The first author interviewed all the participants. Most of the interviews were conducted at the PHNs office, but some interviews had to be conducted via telephone due to the COVID-19 pandemic. Each interview lasted approximately 45 minutes. The interviews were recorded and transcribed by the first author. Both authors participated in the content analysis, which secured consensus.

### Data analysis

The interviews were analysed using qualitative content analysis by Graneheim and Lundman (2004). The text was divided into meaning units, a constellation of words with the same central meaning. Furthermore, the meaning units were condensed, shortening the text but keeping the core meaning, and thereafter given a code. Codes are labels of the meaning unit, and common content was sorted into categories. A category is a group of content that shares a commonality. The categories are the manifest content and the sub-themes and themes occurring as the latent content, meaning the categories represent the descriptive meaning, and the themes represent the interpreted meaning of the text. Themes are created to find the underlying meaning of the text (Graneheim & Lundman, 2004). The themes are described by Lindgren et al. (2020) as a “red thread” throughout the analysis, including the meaning of the phenomenon. Table I shows the interpretation process from codes, categories, sub-themes, and themes. The analysis process was a collaboration between both authors by discussing the findings back and forth and then achieve consensus of suitable codes, categories, sub-themes, and themes.

**Table I.** Example of the development from meaning unit and condensed meaning unit to code, category, sub-theme and theme.

Meaning unit	Condensed meaning unit	Code	Category	Sub-theme	Theme
*It is important for me to know that I am not alone in this follow-up*	Important not feeling alone	Alone	Interdisciplinary collaboration	PHNs feel alone with the responsibility to follow up with children with overweight and obesity.	PHNs call for clearer guidelines for the follow-up of children with overweight and obesity.
*The GP often does not inform the PHN about their assessment, and there is rarely any collaboration there*	PHN lack information from GPs about their assessment	GPs behavior to child obesity	Interdisciplinary collaboration		
*I think it can sometimes be difficult because the parents can come back from the GP and say that everything has been checked and that the doctor thought it was strange that they contacted him. In a way, the GP is not on our team*	Follow up is difficult when the doctor thought it was strange that the parents contacted him- GPs not on PHNs team	GPs attitude to child obesity	Interdisciplinary collaboration		
*I experience that the collaboration with the GP is good. We work closely together. What is positive about a smaller municipality is also that most of the children has the same GP*	PHN work close with GPs in small municipalities where most children have the same GP	Collaboration with GPs	Interdisciplinary collaboration		
*I expect the child’s weight trend to reverse when we refer them to the specialist health care, but I rather experience that the children continue to have an increase in weight*	PHN expect referral to specialists would reverse weight trend, but experienced the opposite	Specialist health care	Interdisciplinary collaboration		
*I wish they could call me and hear what I have done and thought*	PHN wish specialists would be interested in her view	Specialist health care	Interdisciplinary collaboration		
*…, but I think the guidelines are too rigid. I think they are too strict in relation to how the average body is or how the average Norwegian looks like*	Strict average body weight makes the guidelines too rigid	Unclear national guidelines	Rigid national guidelines	PHNs have suggestions to clarify the national guidelines.	
*Even if a child ends up at overweight, but has been there stable for a long time, then I think I have worked long enough to see with my inner clinical view that I do not need to call the parents*	Clinical experience helps to assess when to call the parents if the child’s overweight has been stable for a long time	Use clinical judgement	Clinical judgement		
*What does the research say about involving a child in lifestyle changes? Maybe it should be something about it in the guidelines? I think a revision is needed to ensure and clarify how we involve the child in the follow-up*	A revision of the guidelines is needed to ensure and clarify how to involve the child in the follow-up	Missing in the guidelines	Guidelines need to be revised		

### Ethical considerations

The interviewer was aware that it could be sensitive for the PHNs to talk about their experiences of following up children with overweight and obesity. As mentioned in the introduction, the work with children with overweight and obesity that PHNs are responsible for, is controversial in the society, which can contribute to uncertainty as to whether PHNs are doing good or good enough. The PHNs being interviewed may have personal thoughts and experiences regarding obesity that may evoke emotions. It might be difficult for the participants to share their experience, and therefore it was important for the interviewer to show interest, listen actively (Dempsey et al., 2016), and show appreciation for the information the PHNs shared. The interviews were well planned, with an interview guide moving from broader issues to more specific and sensitive issues.

The Norwegian Centre for Research Data (NSD) approved the study by number 878,733. The participants received written information about the project, including the aims, methods, and how we would ensure privacy and confidentiality for the participants. The participants were informed of the possibility of withdrawing from the study. Written consent from all participants were gathered and stored securely with no possibility of connecting to the interviews. The interviews were securely stored using the application “Nettskjema”, which stores data in an encrypted and safe form (Universitetet i Stavanger, 2021). All recordings were deleted after transcription.

We have submitted the study to Regional committee for medical and healthcare research ethics (REK), which have assessed that the project does not require an application, number 655,889. This is due to the material not containing data about human health and illness, but rather experiences from health professionals within the health service.

## Results

Nine participants were interviewed, eight of whom were educated PHNs. One participant worked as a PHN without the formal education but was a registered nurse. This participant was included in the study because she provided valuable information. Furthermore, registered nurses working as PHNs, despite the lack of formal education, are not uncommon in Norway due to challenges in recruiting educated PHNs. The registered nurses have the same tasks as those who are educated as PHNs regarding the follow-up of children with overweight and obesity. The participants had different work experiences, ranging from 2–25 years. All of them worked as a PHN in the primary school within five municipalities in Western Norway. The PHNs were engaged when talking about overweight and obesity amongst children in primary school, and several of the PHNs were glad to have the opportunity to talk about the topic.

During the analysis, two main themes and five sub-themes emerged, as shown in Table II. Prominent findings in the interviews included the PHN’s strong feeling of responsibility to follow up with children with obesity and their families; at the same time, they experienced several challenges. National guidelines were important but, for many reasons, difficult to use. The PHNs were highly concerned about serving this vulnerable group of children, and they tried to meet their needs. However, encounters and conversations on weight were difficult because of the various and challenging emotions the discussion evoked. Another prominent finding was that PHNs need the national guidelines to be a clearer tool to help them offer better service for children with obesity. The PHNs felt alone in the responsibility of the follow-up of the children and their families. They called for a stronger commitment to sharing responsibility between professionals and clearer guidance for carrying out interventions. The PHNs had several recommendations to make national guidelines clearer.

**Table II.** Overview of themes and sub-themes.

Themes	The important but challenging duty of following up with children with overweight and obesity			The PHNs call for clearer guidelines for the follow-up of children with overweight and obesity	
Sub-themes	The PHN strives to adhere to the national guidelines	The PHNs show compassion in the follow-up of children with overweight and obesity	The difficulties in handling parents’ feelings and reactions in weight conversations	The PHNs feel alone with the responsibility to follow up with children with overweight and obesity	The PHNs have suggestions to clarify the national guidelines

### Theme 1: the important but challenging duty of following up with children with overweight and obesity

The PHNs experienced that they had an important role in helping children with overweight and obesity and their families. They found the guidelines to be important tools when assessing a child’s weight development. Guidelines were also considered important to offer equal health services for all children. PHNs strived to meet children and their caregivers in a careful manner and emphasized the importance of good relationships with the families. When using the guidelines, PHNs met challenges such as difficulties communicating a child’s weight to parents and how to deal with negative feedback from parents. The PHNs reported to use different communication strategies to overcome barriers of informing parents about their child’s overweight.

### The PHN strives to adhere to the national guidelines

It appears that the PHNs want to follow the national guidelines and do the expected tasks. The PHNs are concerned with the quality of the service and that the follow-up provided by the PHNs should be in the best interests of the child. Through the interviews, it was perceived that the PHNs take responsibility and initiative in the follow-up of childhood overweight and obesity.

All the PHNs interviewed follow the height and weight measurements in the guidelines, but the services offered in the different municipalities vary. This seems to be related to the size and resources of the municipality. Several municipalities offer courses to parents about healthy diet, and some also arrange activities for the children. Some municipalities offer individual follow-up in addition to the regular follow-up by the PHN. This “extra” follow-up seems to be given by a PHN or a physiotherapist. In the smaller municipalities, there are fewer such offers, but it seems to be a clearer and more solid collaboration with the GP.

One municipality is in the process of offering courses to parents on diet but also including parts of the COS-P-course (circle of security), which could support parents in strengthening the relationship with their child. The PHNs from the smaller municipalities said they could address the topic of overweight and obesity on a system level. An example of this could include discussing the availability of unhealthy food items in public schools with other professionals. The PHNs said this is done to see if it is possible to prevent obesity by engaging at an interdisciplinary level. In this way, they also focus on health promotion, a central aspect of the PHN role.

But I can address that I for example observe that there is a growing tendency, that more children are overweight or obese, and ask them what they think about it. Then I address overweight more on a general level and try to lift it up to see how to prevent in a higher and more effective way. (informant 8)

PHNs try to follow the advice given in the guidelines, and most of the PHNs consider their work dealing with overweight and obesity as important for children’s health. They measure the children in first and third grade and follow up with those who are overweight and obese.

Actually, I feel like this is an exciting and educational task to do. It is important because it involves the child’s health. (informant 2)

Another PHN emphasized the importance of adults teaching children to regulate themselves when it comes to food intake. This PHN considers it important for the child to be able to make good choices for their health later in life.

Children can eventually learn to regulate themselves, but adults must show it to them first. (informant 6)

Some municipalities have given more flexibility to how the PHNs can communicate the results of the measurements to the parents. Previously, many of the PHNs called all parents of children who were overweight or obese. Now, the PHN in some municipalities can send a letter by post or digitally to parents with information about the result and available follow-up offers, encouraging the parents to get in touch if they want follow-up from the PHN or to participate in available courses in their municipality. The PHNs who have tried this communication method reported that it makes the communication of the result to the parents easier for them by being more time-effective, and they do not get an immediate negative reaction from the parents.

By sending digitally instead of calling, you get to say something you want without getting any “spikes” back. (informant 6)

Another PHN took a critical look at this information method because, in her experience, it is the “resourceful” parents who contact her. This method may lead to the follow-up missing the target group and those who need extra support from the PHN.

A voice among PHNs lately has been that parents themselves can get in touch if they want a follow-up and are worried about their child. By using this method, I think many children that need our help are not detected. I believe it is the resourceful parents who already have the knowledge and good regulation ability who will make the contact. This actually make the differences bigger. It is the parent who already struggle with regulation and struggles to make good habits who also become those who are unable to contact us. (informant 2)

Some PHNs also described socioeconomic status as a factor for parents’ attendance regarding the follow-up of a child’s weight.

I would say that there is a difference between the resourceful families and those who do not have the same capacity. Then I emphasized the importance of parental guidance, and in these cases I may push the parents a little extra. (informant 9)

Some informants meant that they did not use motivational interviewing as a communication method in the follow-up of overweight and obesity due to lack of competence in using this method.

I usually do not use motivational interviewing; I do not have the competence to use it. Sometimes I look at a PowerPoint about motivational interviewing before talking to the parents about a child’s weight, just to boost myself. (informant 6)

In some municipalities, the PHNs are now more concerned with communicating to the parents that the follow-up of overweight and obesity is voluntary. The parents get information about the measurement being voluntary orally and in writing from the PHN. PHNs who clearly communicate this have observed that the parents who say no to measurements or follow-up are often those with children who the PHNs believe would have been overweight or obese.

I have had some parents saying they do not want to measure their child. These children seem to be overweight or obese. (informant 4)

### The PHN shows compassion in the follow-up of overweight and obese children

All PHNs show compassion in the follow-up of overweight and obese children. They are concerned about shielding the child from the results, and emphasize passing the responsibility for the child’s weight development to the parents. Several PHNs also emphasize the importance of building a good relationship with the parents and children. None of the PHNs wants the focus on physical appearance; rather, they aim to emphasize health.

The PHNs are interested in the parents’ and the child’s perspectives. Instead of just giving advice to the parents, many PHNs emphasize listening to their thoughts first and then asking if they want help.

… as I have worked a bit longer as a PHN and become a little wiser, I realized that I must try to tune into where the family is and what they may need. (informant 6)

Several PHNs also said they must respect if the parents do not want follow-up or measurements.

If they say no, I must stay calm until they are ready again. That is not the right time to push them. (informant 3)

In smaller municipalities, the PHNs often know the families and follow the child from birth until they finish primary education. These PHNs have a unique opportunity to build a good relationship with the parents and child over a longer period.

Perhaps it is a bit easier for me because most of the children I have followed since they were born. (informant 9)

None of the PHNs wanted a focus on physical appearance. The PHNs did not want the child to be on a strict diet but guided the parents to focus on healthy food so that, for instance, the child could participate in activities with other children without problems. The PHNs were also concerned about minimizing the risks of illness and health consequences of being obese later in life.

As a rule, I always tell the parents that we do not want to put their child on a diet but focus on the child having a better health. (informant 8)

The PHNs believed it is the parents’ responsibility to facilitate a healthy diet for their child. In addition, they believed that showing the child the results could make the child more aware of their weight and body and compare themselves with others.

I never show the result to the children. I have experienced that if the children get to know the numbers, there will be a lot of comparison. (informant 4)

### The difficulties in encounter parents’ feelings and reactions in weight conversations

Several PHNs described the follow-up as difficult for them. They found it particularly challenging to talk to the parents about the child’s weight. They experienced that parents often reacted negatively to the information provided by the PHN. Especially when calling the parents, the PHN felt uncomfortable and dreaded the conversation.

…, but I do not like these conversations because I have been scolded. I dread every of these conversations. I was so afraid in advance of one conversation, that I postponed it many times. (informant 4)

Some PHNs also said that parents experience them as controlling and believe that the PHN interferes in their everyday life and monitors whether they make the right decisions. This seems to be an uncomfortable position for the PHN to be in.

I almost feel like I am having my hand into to their bowl of chips on a Friday night. I do not want to be that controlling. (informant 6)

Several PHNs said they are afraid that the follow-up may lead to negative consequences for the child in terms of being more aware of their own body and weight and cause lower self-esteem. Many PHNs seem unsure whether involving the child in the follow-up may lead to negative consequences for the child.

But I am afraid that this follow-up may harm the child, that it may contribute to poorer mental health and affect the child’s body image and self-esteem negatively. (informant 4)

Some PHNs were afraid the follow-up may have contributed to the child having negative thoughts about their body. The PHNs believed this may have led some children to lose weight, but in some cases, it may have contributed to the child developing an eating disorder.

I have talked to her so much and understood what kind of body image she is left with, and that some of this body image, not all, but a small drop of it may have been caused by him taken regularly out for measurements. Then it is a shame if these measurements result in a negative body image. Now this child has a disturbed body image and has troubled with eating. (informant 6)

Several PHNs expressed that parents were also unsure whether the follow-up may have negative consequences or harm the child. The PHNs described that some parents are afraid that the follow-up can cause lower self-esteem or that the child may develop an eating disorder due to the extra focus on weight and food.

Some parents may think that it is not good for the child to be measured that often and they are afraid that the child will get upset. It was a mother a few weeks ago that called me and said: “Now my son comes home and asks if he is fat”. (informant 7)

Because of the parents’ scepticism to the follow-up, the PHNs experienced an increased number of parents who said they did not want their child to be measured.

Another year, a parent contacted me and said they did not want their child to be measured because she had a sibling that had an eating disorder. The parent expressed that he was afraid that the other child also could develop an eating disorder. The parents believed that measurement in 3^rd^ grade had contributed to the sibling having an eating disorder. (informant 4)

In all interviews, the PHNs said it is difficult for parents to be informed that their child is measured with overweight or obesity. The PHNs must be careful with how they express this to the parents without upsetting them.

Most parents do not want a conversation or a phone call that their child is overweight and obese. Many gets very upset when I am calling them. (informant 1)

Factors described by the PHNs that may contribute to a positive weight development for the child are that parents share the same concern as the PHN, that the whole family is motivated for change, that the PHN has time and resources to provide an adequate follow-up, and that the PHN is confident in their role.

I also get some positive reactions on the follow-up. I think it is important to have enough time and being able to meet the child and the parents in a good way. I am helped by having a long work experience as a PHN. That makes me confident in the work I do. (informant 3)

### Theme 2: PHNs call for clearer guidelines for the follow-up of children with overweight and obesity

The PHNs meant clearer guidelines would increase the quality of care for children with overweight and obesity and their families. They wanted the guidelines to emphasize interdisciplinary collaboration to a greater extent, and thereby ensure an improved dialogue between health professionals. The PHNs were also unsure whether the definition of overweight should be revised or not. They also requested a description of how and when follow-up may lead to negative health consequences for the child.

### Phns feel alone with the responsibility to follow up with children with overweight and obesity

In several interviews, the PHN expressed a lack of interdisciplinary collaboration. The PHNs experienced challenges in dialogue with the GP, and several PHNs expressed that they miss feedback when the GP has made an assessment.

The GP often does not inform the PHN about their assessment, and there is rarely any collaboration there. (informant 4)

Several PHNs experienced that the GP had a different opinion on the child’s weight and that the GP did not share the same concern as the PHN. This led to the PHN being unsure whether it was the correct assessment to refer the family to the GP in the first place.

I think it can sometimes be difficult because the parents can come back from the GP and say that everything has been checked and that the doctor thought it was strange that they contacted him. In a way, the GP is not on our team. (informant 2)

Several PHNs also mentioned that they missed having someone to share the responsibility and discuss the cases with.

It is important for me to know that I am not alone in this follow-up. (informant 8)

The smaller municipalities differ in terms of their experience of collaboration with the GP. In smaller municipalities, the PHNs usually receive feedback from the GP during and after the follow-up. The PHNs associate this with almost everyone having the same GP, that the PHN and the GP have meeting points, and that they are physically working near each other.

I experience that the collaboration with the GP is good. We work closely together. What is positive about a smaller municipality is also that most of the children has the same GP. (informant 9)

The PHNs also described that collaboration with specialist health care was lacking. They expected a closer and more specialized follow-up from the hospital but experienced that the specialized health care took many similar measures and assessments as the PHNs.

I expect the child’s weight trend to reverse when we refer them to the specialist health care, but I rather experience that the children continue to have an increase in weight. (informant 9)

Several PHNs missed the dialogue and feedback from the specialist health care during and after follow-up. The PHNs said they were not consulted by specialist health care providers on individual cases.

I wish they could call me and hear what I have done and thought. (informant 7)

In general, the interviews expressed a lack of or poor interdisciplinary collaboration in the follow-up of overweight and obese children. Several interviewees mentioned that the various professions are uncertain about their own and other areas of responsibility.

### Phns have suggestions to clarify the national guidelines

Several PHNs thought the guidelines were too rigid or strict. They experienced that healthy and active children who are not visibly overweight are still being classified as overweight by the scale given in the guidelines. The PHNs then debated whether they should call the child’s parents to inform them. The PHNs were unsure whether the limit for overweight should be reviewed.

…, but I think the guidelines are too rigid. I think they are too strict in relation to how the average body is or how the average Norwegian looks like (5)

Maybe the limit for overweight should be reviewed or sat up a bit? (informant 1)

It appears that some PHNs use their clinical judgement when deciding whether to call the parents when overweight is identified. Some of the PHNs interviewed do not contact the parents of the children who have been stable at the same weight (overweight) for a long period of time. The PHN then considers this as the normal weight for the child. They do not call the parents because they do not want to create unnecessary worries for them and the child.

Even if a child ends up at overweight, but has been there stable for a long time, then I think I have worked long enough to see with my inner clinical view that I do not need to call the parents. (informant 3)

Many PHNs said they are unsure whether the follow-up may lead to negative consequences for the child. This is also a concern among parents. Therefore, one PHN wants this to be a part of the guidelines. She wants the guidelines to include research on whether to involve the child in the follow-up of overweight and obesity and how to involve the child in a good and healthy way. This could support the PHN’s work and reassure parents that it will not harm the child if it is done properly.

What does the research say about involving a child in lifestyle changes? Maybe it should be something about it in the guidelines? I think a revision is needed to ensure and clarify how we involve the child in the follow-up. (informant 2)

In all interviews, the PHNs expressed a lack of interdisciplinary collaboration. Therefore, several PHNs want a clearer and better interdisciplinary collaboration described in the guidelines.

I think that it should be written more in the guidelines about the interdisciplinary collaboration between the primary health care and the specialist health care. And that the PHN could receive feedback, preferably once a year or every six months on the plans, what is assessed etc. (informant 7)

Another PHN suggested more cases and examples of how interdisciplinary collaboration could be solved.

Maybe it should be a case on how this could be solved around some children. What does a collaboration meeting contain and where does it take place? How is it done in practice, who takes the initiative, who follows up etc. (informant 6)

## Discussion

This study aimed to develop knowledge of how the follow-up regarding overweight and obesity among children in primary school is experienced by the PHN and how the guidelines may be used to improve health services in this follow-up. The interviews provided information on how the follow-up is experienced by the PHNs and what PHNs believe could contribute to an improved follow-up for the child and family as well as for the PHNs.

The results will be discussed in relation to previous research, the Norwegian national guidelines for the prevention, assessment, and treatment of overweight and obesity for children and adolescents (Helsedirektoratet, 2010), and Hildegard Peplau’s interpersonal relations theory (Peplau, 1991).

Throughout all the interviews, weight was defined as a vulnerable topic for both parents and children, as well as difficult for the PHN because it seems to challenge the PHN’s attitudes. Most of the PHNs experienced a dilemma of following the guidelines by informing the parents about a child’s weight development versus taking into consideration how the parents will react.

The PHNs felt that the parents were sceptical about the follow-up of overweight and obesity. The PHNs described parents feeling controlled and stigmatized and not always sharing the PHN’s concerns about the child’s weight. These findings are consistent with previous research in which PHNs have been interviewed regarding the follow-up of overweight and obesity (Støles et al., 2019; Thorstensson et al., 2018). The same findings are also found in interviews aimed towards the parents’ experience (Toftemo et al., 2013). Ames et al. (2020) also found that most of the parents did not accept the result when their child was measured in the category of obesity because they did not consider their child to be overweight or obese. Many of the parents distanced themselves from the result, which led to parents being less likely to change their behaviour regarding their child’s weight (Ames et al., 2020). This is consistent with findings in another Norwegian study (Melbye & Hansen, 2015), in which parents’ understanding of their child’s weight is crucial to implement measures for their child. This study showed that parental understanding that excess childhood weight may have consequences for the child’s health led to concern, leading to restrictive feeding strategies (Melbye & Hansen, 2015).

According to the findings in this study, parental lack of engagement in the child’s weight is frustrating for the PHNs. The PHNs end up feeling powerless in meeting the requirements given in the guidelines and, at the same time, show consideration for the parents’ reactions. In relation to Peplau’s interpersonal theory, the two factors that lead to a therapeutic alliance—a “felt need” from the patient and that the help sought is considered useful—are not present in this situation. According to Peplau, this means it is impossible to make a good alliance with the client (Peplau, 1991). This may indicate that the PHN is not able to help the family. Parental concern and engagement regarding the child’s weight are crucial for a weight change (Ames et al., 2020; Melbye & Hansen, 2015). Povey et al. (2020) also found that when help was perceived as not needed by the parents, the families disengaged in the obesity management programme. By not considering their child obese, the parents did not see the point of attending the programme (Povey et al., 2020). To be positioned to help the family, how the PHN communicates the result to the parents may be crucial. Nursing goals are often in opposition to the patient’s goals. Still, communication in interpersonal relationships aids both the nurse and the patient in clarifying their goals and reaching a common understanding (Peplau, 1991). Consequently, the PHN should spend time listening to the parents’ goals and feelings.

Each interview asked how the PHN communicated the child’s weight status to the parents. The results show that it is more common to communicate the result digitally or by post, where the parents are encouraged to contact the PHN if they want to receive follow-up or are worried about their child’s weight. The opportunity to use a digital platform (helsenorge) has recently been available in Norway as a secure communication tool between patients and health care providers (Hageberg, 2011). Many of the PHNs in this study who have tried this communication method experienced that fewer parents contacted them after receiving information about the weight result. If the parents do not make contact, the follow-up seems to be ended. One PHN also experienced that it is the “resourceful” parents who contact the PHN, which may contribute to greater differences in access to health care for some children with overweight and obesity. This may lead to not reaching the families who need support the most from the PHN. Thus, the PHNs are not hitting the target group of the follow-up, which in turn might lead to more serious health consequences because obesity might increase in the child. Povey et al. (2020) found that many parents reacted negatively to the letter providing the result of the measurement. The parents were angry even before the follow-up had started and felt judged about their parenting abilities. Communication is key for the receptiveness of the parents, and face-to-face communication may be a better solution than a letter to get in a position to help the parents and the child (Povey et al., 2020).

Other research also found parents being left alone interpreting the results after measurement in the school health service. According to Støles et al. (2019), this may lead to parents interpreting the results negatively, both related to the child’s health and their care for their child (Støles et al., 2019). Ames et al. (2020) found that parents wanted help from health professionals to interpret the results (Ames et al., 2020). The parents need an explanation of the results but do not get it due to the way the PHNs in our study communicate the child’s overweight or obesity. According to Peplau’s interpersonal relations theory, the nurse should offer the patient direction during a current difficulty and supply the patient with knowledge (Peplau, 1991). An important part of the interpersonal relationship is, therefore, not fulfilled. It may be that the PHN chooses to communicate the results using this method due to the lack of time and to avoid negative reactions from the parents. However, digital communication methods should not be used if they result in the PHN not reaching out to those children in need of help to avoid severe health consequences.

Many PHNs also focus on communicating to the parents that the follow-up is voluntary. Several PHNs say that parents who refrain from having their children measured seem to be the cases where the PHN suspects the child to be overweight or obese. This may distort the national statistics on obesity and overweight among children simply because parents do not allow their children to be weighed by the PHN. Peplau emphasizes building a relationship with the patient by active listening and accepting and exploring the patient’s feelings to be in a position to help the patient (Peplau, 1991). This is consistent with recent studies such as (Argelich et al., 2021), who found that when addressing a weight problem with the parents, it is important to do so with respect and active listening without judging, scaring or blaming the parents. This may lead to the parents’ acceptance of the problem (Argelich et al., 2021). When parents say they do not want to measure their child or receive follow-up, the PHN should explore the parent’s concerns and meet the parents’ needs for knowledge, according to Peplau’s role as a resource. Parents may be concerned that talking with their child about obesity might promote an eating disorder or lower self-esteem. According to Peplau, the PHN should act as a guide and give factual information that the risk of an eating disorder is not imminent when the child receives professional help to change habits. Structured and professionally run obesity treatment was associated with reduced prevalence, risk, and symptoms of eating disorders (Jebeile et al., 2019).

According to the national guidelines, the prevention of overweight and obesity requires effective systems for interdisciplinary collaboration (Helsedirektoratet, 2010). According to Clancy et al. (2013), trust, respect, and competence in interdisciplinary collaboration are important success factors for collaboration between different professions (Clancy et al., 2013). Therefore, interdisciplinary collaboration also involves interpersonal relationships between professionals. The importance of trust and respect is consistent with Peplau’s interpersonal relations theory, in which trust and respect are two key factors for building a good relationship (Peplau, 1991). However, there seems to be a lack of interdisciplinary collaboration among the PHNs being interviewed. The PHN is responsible for initiating an interdisciplinary collaboration, but when reaching out to the GP, the PHN experiences no dialogue or feedback, or in some cases, the GP does not share the PHNs concern. This may result in the PHN feeling a lack of trust and respect for the GP and may affect the collaboration. The same lack of interdisciplinary collaboration is also experienced with the specialist health care. Interdisciplinary collaboration and organizational embedding were the determinants identified that most frequently affect the implementation of the Norwegian national guidelines for handling childhood obesity (Nordstrand et al., 2016). The absence of cooperation with other agencies is also found in other European countries (Brüggen et al., 2020; Isma et al., 2013).

Organizational factors are also seen as barriers to obesity prevention. Primary care providers’ beliefs that organizations are lacking in their efforts may have contributed to their sense of futility with regard to the potential impact of obesity prevention efforts (Ray et al., 2022). According to Akselbo and Ingebrigtsen (2015), mothers of overweight and obese children receiving treatment for obesity in specialist health care experienced no further follow-up in primary health care (Akselbo & Ingebrigtsen, 2015). This may be due to a lack of communication between primary and specialist health care and may represent a deficiency that can affect children with obesity’s health. The PHNs in this study also experience various professions having little competence about each other’s responsibility in interdisciplinary collaboration. According to Dahl and Crawford (2018), mutual understanding of each other’s competence is crucial to establish good interdisciplinary collaboration (Dahl & Crawford, 2018).

However, the PHNs from the smaller municipalities in this study experienced a better collaboration with the GP and associated this with, among other things, physical proximity. According to Clancy et al. (2013), physical proximity is important to establish a successful interdisciplinary collaboration. Physical proximity may lead to informal discussions that may help to establish relationships with other professionals and lead to professionals being more likely to be open to other points of view in informal settings. The study, therefore, shows that municipality size may influence interdisciplinary collaboration (Clancy et al., 2013).

The PHNs experience the guidelines to be rigid and insufficient. They experience that many children who did not appear to be overweight still measured as overweight after first and third grade. This is consistent with previous research (Helseth et al., 2017). The PHNs decided not to contact all parents of overweight children. They explained that they used clinical judgement and did not contact the parents of those children whose weights were stable over time. According to the guidelines, BMI alone is not a sufficient indicator of being overweight or obese, and health professionals must use it together with other findings (Helsedirektoratet, 2010). This is also consistent with recent research by Madsen et al. (2021), who found that BMI screening alone does not improve the child’s health and should not be done without effective interventions available for the child and the family (Madsen et al., 2021). Earlier research on implementing the Norwegian guidelines found different views from public health nurses (Nordstrand et al., 2016). Pragmatic PHNs considered the guidelines a useful source of knowledge, critical PHNs called for more concrete answers in the guidelines, and resigned PHNs were not very familiar with the new guidelines; therefore, they worked with childhood overweight and obesity in the same way as before the guidelines were established (Nordstrand et al., 2016). However, Dahl and Clancy (2015) stated that guidelines might lead to health professionals focusing more on the tasks and expectations given in the guidelines rather than on clinical judgement. Excessive use of guidelines and protocols may lead to the instrumentalisation of the PHN’s role (Dahl & Clancy, 2015). At the same time, a recent study shows that school nurses in Sweden do not have official guidelines and that school nurses miss a mutual structure on when and how to act regarding overweight and obesity. It was difficult for the school nurse to meet the parents without an evidence-based national strategy. The school nurses considered this important to ensure equality and quality in the health service (Gothilander & Johansson, 2021). The guidelines are therefore necessary, but the PHN’s clinical judgement is also crucial in detecting if a child is overweight or obese.

## Methodological considerations

The concepts of credibility, dependability, and transferability are used to assess the study’s trustworthiness (Graneheim & Lundman, 2004). Using semi-structured individual interviews with an interview guide was suitable for exploring the PHNs’ experiences and met the aims of the study. With a hermeneutic-phenomenological approach using an acknowledged analysis method—content analysis by Graneheim and Lundman (Graneheim & Lundman, 2004)—it was possible to explore the PHNs’ experiences and, at the same time, interpret the content given by the PHNs. Individual interviews were chosen to get a detailed description of the PHNs’ experiences and allow sufficient time for each participant. The participants’ descriptions of experiences regarding the follow-up of overweight and obesity were answered by the researcher with open-ended follow-up questions. To ensure that no new and crucial information about the PHNs’ experiences was left out, the number of participants was increased until the interviews repeated much of the same information. Having participants with different work experiences as PHNs also contributed to the credibility of the study. The analysis was done in collaboration between both authors, sharing thoughts and having a dialogue regarding preunderstandings, meaning units, and creating appropriate themes. This ensured consensus.

Dependability involves whether the work process in your study is visible to the reader. The reader should be able to understand and see how the researcher ended up with the results of the study (Nielsen et al., 2021, p. 283). This study was focused on having a clear distinction between citations from the participants and the researcher’s own interpretations.

Transferability involves whether the result from the study may be transferred to other settings or groups (Graneheim & Lundman, 2004). A qualitative study meets the criterion of transferability if individuals not involved in the study can associate the results with their own experiences (Cope, 2014). Participants were gathered from one county in Western Norway, and it was a difficult recruitment process with few PHNs reaching out. Snowball sampling was therefore used, which may result in the participant sample not being random. For instance, the participants may have similar characteristics, which could interfere with the transferability (Sadler et al., 2010). Nevertheless, much of the information gathered from the participants in this study is similar to findings in other studies regarding overweight and obesity in children. In addition, the various municipalities were of different sizes, which may give a variety of experiences more transferable to other settings and groups.

Throughout the study process, we discussed how many informants were needed to fulfil information power, as described byMalterud et al. (2016). Due to the relatively narrow aim of the study, interviews with 7 − 9 PHNs were initially considered appropriate. Through heads of the school health service departments and by snowball recruitment from already included informants, we managed to recruit participants holding characteristics that were highly specific for the aim of our study. The estimated number of participants was also based on experiences from previous research on the topic. The participants spoke freely, were engaged, and gave rich and diverse narratives of their experiences. We therefore considered that the few participants offered sufficient information power to the study (Malterud et al., 2016).

## Implications for practice

The guidelines should be clearer and include how the public health nurse can communicate to parents about their child’s weight status in the best possible way. One-way communication should be avoided. If they avoid talking to the parents about the result, the public health nurses are unable to give guidance to those families in need of it, and the parents’ capacity may determine whether a child receives help from the PHN. Motivational interviewing is recommended in the guidelines as a conversation tool when talking to parents about lifestyle changes, and resources for educating PHNs should be prioritized by the municipalities’ management.

A clearer interdisciplinary collaboration could also improve the follow-up, but it must be an expectation that the health professionals prioritize this group and have meeting points, as well as give feedback to each other during the follow-up. Overweight and obesity must therefore be on the agenda by leaders of the school health service and politicians, which must facilitate the necessary resources and an increase in competence for the PHNs regarding childhood overweight and obesity.

Public health nurses must speak up about their needs in the school health service and their lack of resources in order to contribute to the follow-up of children with obesity. This may be done by reporting deviations in the school health service.

Public health nurses are uncertain about how to communicate with parents about overweight and obesity in children. Further research should include parents’ experiences of conversing with the public health nurse about overweight and obesity to attain the parents’ perspective. It should also further explore the public health nurse’s role in helping families prevent childhood obesity. Further research should also include which resources are required in the school health service to achieve a sufficient follow-up for overweight and obese children.

## Conclusion

Public health nurses are strongly aware of their responsibility laid down in national guidelines for follow-up of children with obesity. They are highly motivated to care for the children and their families but experience several obstacles to implementing a sufficient quality follow-up. A main challenge for the public health nurse is to get in a position to help the parents due to misunderstandings between the parent and the PHN on whether obesity in the child requires further followed-up with interventions. Parents’ obstacles included fear of eating disorders. Sending letters to parents about the child’s weight saved time, and then the PHN did not have to face the parents’ feelings. This praxis led to PHNs’ concerns that some children who need follow-up do not receive it, which could result in inequality in health. PHNs should listen to the parents’ concerns and feelings and try to understand their perspective, as Peplau emphasizes in the role of a stranger. It is crucial for the PHN and the families to establish a common understanding and goals. PHNs should avoid one-way communication and meet the parents’ concerns and needs properly by taking on the roles of resource, teacher, surrogate, and counsellor, as described by Peplau. Other obstacles for PHNs were the lack of common understanding between different disciplines, lack of cooperation between the municipal and specialist health care services, and lack of resources to follow up with children with obesity.

PHN should make known what is perceived as unclear in the guidelines, and deviation from following up according to the guidelines should be reported. According to Peplau, PHNs should act as leaders and task the municipalities’ authorities to take responsibility for promoting necessary interdisciplinary collaboration and ensuring adequate resources.
